# The effectiveness of case management for comorbid diabetes type 2 patients; the CasCo study. Design of a randomized controlled trial

**DOI:** 10.1186/1471-2296-12-68

**Published:** 2011-07-05

**Authors:** Nathalie Versnel, Laura MC Welschen, Caroline A Baan, Giel Nijpels, François G Schellevis

**Affiliations:** 1 NIVEL, Netherlands Institute for Health Services Research, P.O.Box 1568, 3500 BN Utrecht, The Netherlands; 2 EMGO+ Institute for Health and Care Research, VU University Medical Centre, Amsterdam, The Netherlands; 3 Department of General Practice, VU University Medical Centre, v.d. Boechorststraat 7, 1081 BT Amsterdam, The Netherlands; 4 Centre for Prevention and Health Services Research, National Institute of Public Health and the Environment (RIVM), P.O.Box 1, 3720 BA Bilthoven, The Netherlands

## Abstract

**Background:**

More than half of the patients with type 2 diabetes (T2DM) patients are diagnosed with one or more comorbid disorders. They can participate in several single-disease oriented disease management programs, which may lead to fragmented care because these programs are not well prepared for coordinating care between programs. Comorbid patients are therefore at risk for suboptimal treatment, unsafe care, inefficient use of health care services and unnecessary costs. Case management is a possible model to counteract fragmented care for comorbid patients. It includes evidence-based optimal care, but is tailored to the individual patients' preferences.

The objective of this study is to examine the effectiveness of a case management program, in addition to a diabetes management program, on the quality of care for comorbid T2DM patients.

**Methods/Design:**

The study is a randomized controlled trial among patients with T2DM and at least one comorbid chronic disease (N = 230), who already participate in a diabetes management program. Randomization will take place at the level of the patients in general practices. Trained practice nurses (case managers) will apply a case management program in addition to the diabetes management program. The case management intervention is based on the Guided Care model and includes six elements; assessing health care needs, planning care, create access to other care providers and community resources, monitoring, coordinating care and recording of all relevant information. Patients in the control group will continue their participation in the diabetes management program and receive care-as-usual from their general practitioner and other care providers.

**Discussion:**

We expect that the case management program, which includes better structured care based on scientific evidence and adjusted to the patients' needs and priorities, will improve the quality of care coordination from both the patients' and caregivers' perspective and will result in less consumption of health care services.

**Trial registration:**

Netherlands Trial Register (NTR): NTR1847

## Background

With the ageing of the population and the increase of prevalence rates of most chronic diseases at older age, increasingly more patients with a chronic disease also suffer from other chronic diseases [1]. In the Netherlands 25-50% of people with a chronic disease is diagnosed with one or more comorbid disorders [2,3]. For patients with type 2 diabetes (T2DM) this percentage is even higher, approximately 60% [4].

Comorbid type 2 diabetes (T2DM) patients can participate in several single-disease oriented disease management programs. Disease management programs are defined as 'systematic, population-based approaches to identify persons at risk, intervene with specific programs of care and measure clinical and other outcomes'. The main goal of disease management programs is to provide good quality care for a chronic disease and to guarantee a central role for the patient in managing the disease [5].

However, participating in multiple single-disease oriented programs in combination with regular primary care, may lead to fragmented care. In designing these programs insufficient attention is paid to the possible comorbid conditions which co-exist with an (index-) disease. Comorbid patients are therefore at risk for suboptimal treatment, unsafe care, inefficient use of health care services, unnecessary costs and consequently run higher risks for adverse events [3,6]. Case management is a possible model to counteract fragmented care for comorbid patients. It is an individualized care program which coordinates all care involved for patients enrolled in different single-disease management programs, who have to adhere to various treatment protocols. It draws on evidence-based optimal care for systematically managing all existing conditions in a patient, and is tailored to the individual patients' preferences [7,8].

There are promising results in a primary care setting from pilot tests on the effects of case management, in which an improvement of the quality of chronic care was observed, especially in the communication, goal setting, decision support, coordination among providers, and in reducing health care costs [8-12]. Recently, a study on the effectiveness of case management in the USA has shown improvement on the self-reported quality of health care for comorbid elderly [13].

To improve the care for comorbid T2DM patients in the Netherlands, we used the Guided Care Model (GC) to design a case management care program customized to the Dutch primary care setting. In the Netherlands, every resident is listed in a primary care practice. The General Practitioner (GP) acts as gatekeeper to secondary (specialized) care which is only accessible after referral. Our aim is to create a comprehensive tool for the general Practice Nurse (PN), which allows her to manage and coordinate the care the comorbid patient needs and to stimulate involvement of the patient in his care process. In this study, the PNs will be trained in conducting the case management program and will hereafter be called *case manager*.

### Theoretical framework

GC is developed in the USA and is based on the Chronic Care Model, a framework to guide quality improvement and disease management activities in the care for patients with a chronic disease [10]. GC combines the elements of the Chronic Care Model in practice and is designed to improve the quality of care and the quality of life for patients with multiple chronic conditions and complex health care needs [7,10].

GC consists of the following elements: assessing health care and well-being needs, communicating intervention possibilities, goal setting and decision support, monitoring and timely evaluation with the patient [10]. These elements intervene on three levels in the patients' care system, on the organizational or practice level (by using the above mentioned elements), on the level of the care provider (by using decision support), and on the level of the patient (by encouraging self management).

### Objective

The objective of this study is to establish the additional value of case management superposed on a diabetes management program, in terms of perceived quality of care, quality of care from the perspective of the GP, the health status of the patient, diabetes control, and health care utilization in T2DM patients with comorbidity.

## Methods/Design

### Design of the study

This study is a randomized controlled trial. T2DM patients with at least one comorbid chronic disease will be assigned to the control or intervention group. All patients participate in a diabetes management system (the Diabetes Care System (DCS), receiving structured diabetes care. In addition, participants in the intervention group will receive the case management intervention. The duration of the intervention is 12 months. The Medical Ethics Committee of the VU University medical centre in Amsterdam approved the study. Participants are allowed to enter the study after signed informed consent. Both the diabetes management program (as part of the usual care in the control group) and the case management intervention will be provided in the practice in which the patient is listed.

#### Description of the Diabetes Care System (DCS)

*The Diabetes Care System (DCS) *is a diabetes management program, which started in 1996 in the West-Friesland region of the Netherlands. By incorporating the Chronic Care Model, the system aims at providing comprehensive regional diabetes care, educating diabetes patients and supporting GPs in treating these patients. By now, nearly 6,500 T2DM patients are registered at the DCS. All receive an annual extended diabetes check-up at the specialized DCS additionally to the diabetes care by their GP according to the guidelines of the Dutch College of General Practioners [14]. The basic principles of the DCS are: the coordination of diabetes care, feedback to the GP and patient empowerment.

Each patient is invited annually by the DCS for a physical examination and diagnostic tests such as blood and urine analysis. In addition, the patient visits a diabetes nurse and a dietician for information and advice in order to improve the skills and confidence needed for self management. The patients are invited for follow-up visits when necessary.

The DCS coordinates the diabetes care between primary and secondary care. Using a centrally organized database, clinical information of patients is accessible to involved health care providers. Results of the annual examination are sent to the patient's GP who is responsible for the management of the patient, delegation of tasks to the PN and the 3-montly follow-up [15,16]. The GP receives feedback from the DCS based on the average results of his patients compared to all patients participating in the DCS.

### Study population

#### Practices

Practices delivering diabetes care in cooperation with the DCS are eligible for participation in this study if they employ a PN for diabetes care and are willing to house the case manager.

#### Patients

Eligible T2DM patients participate in the DCS and are known with at least one of the following comorbid conditions: chronic ischemic heart disease [angina pectoris, previous myocardial infarction, and heart failure], stroke, depression, rheumatoid arthritis, osteoarthritis of hip and/or knee, cancer, chronic obstructive pulmonary disease. The case manager will identify eligible patients from the electronic medical record based on ICPC-codes (International Classification of Primary Care) representing the included comorbid conditions (*see *table 1). Patients are allowed to enter this study when there is not already a case manager involved, when they are not suffering from a health problem which could lead to death within one year, are capable to give informed consent, to personally fill in questionnaires and have sufficient knowledge of the Dutch language.

**Table 1:** International Classification of Primary Care codes of comorbid conditions.

Condition	Code										
**Cancer**	A79	B72	B73	B74	D74	D75	D76	D77	N74	R84	R85
	S77	T71	U75	U76	U77	W72	X75	X76	X77	Y77	Y78
**Chronic ischemic heart disease**	K74	K75	K76								
**Chronic Obstructive Pulmonary Disease**	R95										
**Depression**	P76										
**Osteoarthritis of hip and/or knee**	L89	L90									
**Rheumatoid arthritis**	L88										
**Stroke**	K90										

This table provides an overview of the ICPC-codes corresponding the included comorbid conditions.

ICPC: International Classification of Primary Care

### Randomization

**Figure 1:**
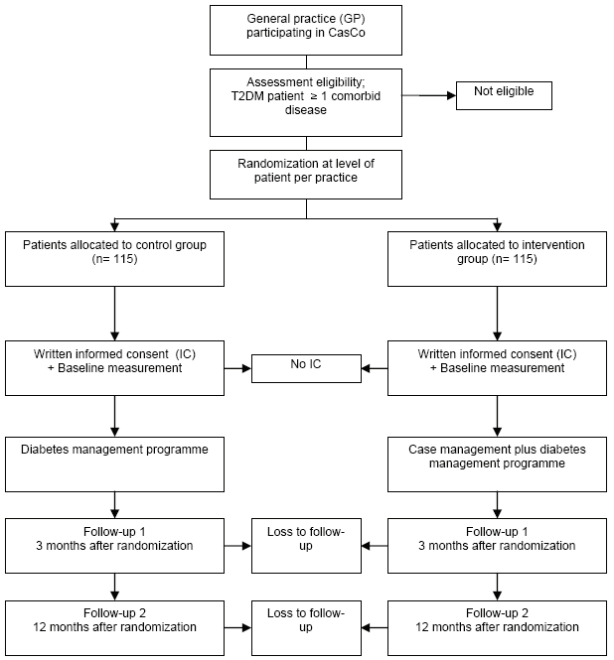
**Design of the study**. The figure shows the design of the CasCo-study.

Patients will be randomly assigned to the intervention or control group (*see *Figure 1). Patients in the control group will receive care from the DCS, their GP and PN. Patients in the intervention group will receive the case management intervention from the case manager in addition. All eligible patients in the participating practices will be invited to join the study. Reasons for non participation will be carefully registered.

### Intervention

The case management program is designed to complement the diabetes management program and therefore is additional to the care from the DCS. The program involves six basic elements from the GC model intended to improve the quality of care for the patient, their access to care, and their capacity for self care, including assessing health care needs, planning care, create access to other care providers and community resources, monitoring, coordinating care and recording of all relevant information [10].

### Training

To execute the program properly, the case manager will be trained in assessing the patients' health care needs (step 1) and in creating a care plan based on the assessment (step 2). The case manager will have frequent contacts with the patient, GP and other care providers. Therefore the case manager will also receive training in motivational interviewing techniques and communication strategies, during a 4-day course and two coaching-on-the-job sessions from a trainer skilled in communication in health care. MI is a patient-centered counseling approach and is intended to help the patient make decisions, to facilitate the patient's participation in care, and to reconsider the patients' priorities with the patient. This method aims at collaboration between the case manager and the patient, using the patient's intrinsic motivation (willingness and motivation for self-care) and care for their autonomy [17,18]. Furthermore, recognizing and using different communication strategies in conversations will help the case manager in their contacts with the patient, GP and other care providers. Coaching-on-the-job will be executed by the trainer during two consults between the case manager and a patient. The trainer will provide feedback following each consult.

#### Description of the intervention: the Case Management Program

The case management program as applied in this study entails the following steps;

##### Step 1) Assessing the patients' health care needs

Before the assessment takes place, the case manager collects information about the patients' co-morbidities, treatments and advices, health care utilization and medication use in the electronic medical records. The initial assessment takes place with the patient in a face-to-face intake at the general practice, by using the Resident Assessment Instrument-Community Health Assessment (RAI-CHA). The RAI was originally designed as a minimum data set to assess the health status of nursing home residents by identification of problems in 18 areas that may need specific care planning [19]. The RAI-CHA provides a comprehensive overview of the patients' medical, functional, cognitive, affective, psychosocial, nutritional and environmental status. The identified problem areas guide the design of an individualized care plan with the intention to improve or maintain the functional health status.

##### Step 2) Start planning care

Together with the GP and the PN responsible for diabetes care, the case manager creates a provisional care plan. This plan is based on information from the assessment and currently provided care by the GP and PN. In case the patient's health problems results in complex care according to the GP, a multidisciplinary consultation with all relevant care providers will take place. Regular participants are the GP, PN, case manager and a representative of the DCS (a dietician or diabetes nurse).

##### Step 3) Discussing the interim care plan with the patient

In a second consultation, the case manager will discuss the provisional care plan with the patient. The case manager will give sufficient information as a result of which the patient will be able to make decisions related to care or managing the problems found. Also, the case manager will inform the patient about community resources relevant to the patients' needs. This results in a care plan suitable to the priorities and needs of the patient and becomes definite after a final review from the GP, PN and case manager.

The care plan includes self-management activities for the patient by setting goals in an action plan (e.g. regarding nutrition, physical exercise, use of medication, and self-monitoring) tailored to the patients' capacities.

##### Step 4) Communicating the care plan to all involved care providers

The care plan will be communicated to the involved care providers in order to coordinate the care. The case manager will not take over the care provided by the other care providers, but will inform them about the patient's care plan and explain the case manager's role.

##### Step 5) Monthly contact with the patient

The case manager will monitor the patient monthly by telephone to detect and address emerging problems regarding the implementation of the care plan and the patients' health status. If necessary, the case manager discusses these problems with the GP and takes appropriate action.

The case manager will review the patient's action plan and stimulate self-management by coaching the patient in achieving goals. The case manager will regularly update the patients' status and care plan using the notes from these contacts.

##### Step 6) Recording

During the intervention period, the case manager will record relevant changes in health status and all actions undertaken in the patients' electronic medical record.

### Control group

The patients in the control group continue participating in the DCS. In consequence, the care patients receive in this group is more extensive than usual care alone. They receive extended diabetes care from the DCS and usual care for diabetes (and the comorbid conditions) from their GP and PN. Care in this group may include the involvement of a PN, referral to other health care professionals, medication reviews, etcetera. These activities will be monitored and measured as part of our data collection. However, these patients do not participate in the case management program and therefore will not be supervised by the case manager.

### Measurements

Table 2 provides an overview of all measurements. Data will be collected using the patients' electronic medical record, postal questionnaires, and semi structured interviews.

**Table 2:** Measurement scheme.

Variable	Instrument	baseline	3 months	12 months
**Primary outcome**				
a. quality of care perceived by patients	CQ-Index GP care PACIC	X X	X X	X X
**Secondary outcomes**				
b. quality of care perceived by GP	Quality Indicators			X
c. health status of the patient	SF-12	X	X	X
d. diabetes control	Hba1c (%)	X	X	X
e. health care utilization	Contacts with care providers	X	X	X
	Medication use (RAI-CHA)	X		X

**Feasibility**	Semi-structured interviews			X

This table provides an overview of the time schedule of all measurements and measures.

CQ-Index for GP care: Consumer Quality Index for GP care; GP: General Practitioner; HbA1c: glycated haemoglobin level; PACIC: Patient Assessment of Chronic Illness Care; RAI-CHA: Resident Assessment Instrument - Community Health Assessment; SF-12: Short-Form 12.

To get more insight into the factors favoring and hampering the implementation of the case management program, we will perform a feasibility check using semi-structured in-depth interviews and collect data via patient questionnaires.

We will interview four groups: 1) all case managers delivering the intervention, 2) the GPs in the participating practices, 3) the diabetes nurses of the DCS, as well as 4) a random stratified selection of 15 patients of the intervention group (both patients who indicated in their questionnaire having been exposed well and poorly to the case management program). The interviews will be recorded using a voice recorder.

### Outcome assessment

Primary and secondary outcome measurements are assessed at baseline, at 3 and 12 months. Interim analyses will be carried out three months after the baseline measurement. This analysis will be purely descriptive concerning changes in the quality of the received care according to the patient as a result of participation in the case management program.

#### Primary outcome measures

The quality of care as perceived by the patient, will be measured with the experience part of the multidimensional questionnaire Consumer Quality Index for GP care (CQ-Index)[20]. The CQ-Index is a standardized systematic way of measuring, analyzing and reporting clients' experiences in care. The 'tailored care' subscale includes 4 items, to be scored on a 4-point scale and has been shown to be reliable (Crohnbach's alpha = 0.84)[21].

Additionally we will use the Patient Assessment of Chronic Illness Care questionnaire (PACIC), a 20-item questionnaire which measures patient-reported quality of chronic illness care. It can be used to determine whether the received care is patient-centered, proactive, planned and includes collaborative goal setting; problem-solving and follow-up support [22].

#### Secondary outcomes measures

The quality of care perceived by the GP will be measured using a validated set of basic quality indicators [23,24]. Quality Indicators define the degree to which the provided care is in accordance with the evidence-based guidelines. They are usually calculated as percentages based on patient data registered in the electronic medical record and from completed questionnaires. Additionally, the Primary Care Assessment Tool provider survey (PCAT) will be used in a semi-structured interview to measure the quality of care from the perspective of the GP as well. The PCAT provides information on structure and process elements of primary care. This also includes information on patient-, provider-, and facility-related perspectives on the experiences of care received and care provided [25].

The health status of the patient will be assessed by the Short Form 12 (SF-12). This is a multi-purpose generic health status questionnaire, including 12 items scoring on a physical and a mental health scale.

Diabetes control will be measured by means of the glycated haemoglobin level (HbA1c). The HbA1c indicates the glucose control over the past 2-3 months and will be measured at baseline and after 12 months.

Health care utilization will be measured by the number of visits patients make to different health care professionals and changes in the medication lists. This information will be collected through a patient questionnaire which was previously used in the second Dutch National Survey of General Practice [26], adapted from the Health Questionnaire of Statistics Netherlands [27]. The questions include involvement of all relevant health care provided. Changes in the medication lists will be measured by comparing the patients' medication lists at the beginning and end of the intervention period.

### Sample size

The required sample size for this study is calculated on the basis of the main outcome measure, the 'tailored care' subscale of the CQ-Index. Previous research showed mean baseline scores of 3.1 (SD 1.0) on a 5-point scale [20]. We expect a mean improvement of 0.4 in the intervention group and 0.1 in the control group (mean difference in improvement 0.3). Assuming a pooled SD of change of 0.75 (SMD = 0.40), we would need complete data for 98 patients in each group; given 80% power and a significance level of 5%. We will recruit 2 × 115 patients allowing a drop-out rate of 15% during follow-up.

### Analyses

The effectiveness of case management will be established in multivariate multilevel analyses, according to the intention-to-treat principle. When possible, specific attention will be paid to identify subgroups of patients characterized by their profile of comorbidity in which the intervention is more effective than in other subgroups. The data might allow calculating an individual case management exposure level score, in the intervention group as well as in the control group. This will enable analyses of the outcome measures according to the per-protocol principle. Differences between patients exposed to the case management program and patients in the control condition on patient's health care utility and patient's health status will be tested using a chi-square test and student t-test, respectively. Potential confounding is checked, including the effect of different case managers and the number of patients per case manager.

## Discussion

In this paper we describe the design of a randomized controlled trial of a case management program led by a trained PN, the case manager, for comorbid T2DM patients. The project will represent a first step in the evidence about the effectiveness of a primary care based case management program by studying the effectiveness in the Dutch primary care setting of a program which has been proven feasible and acceptable in the USA. By superposing this case management program on a diabetes management program, we will also be able to identify its additional value compared to a single disease oriented management program.

We expect that better structured care based on scientific evidence and adjusted to the patients' needs and priorities can improve the quality of care coordination from both the patients' and caregivers' perspective and can result in less consumption of health care services. Patients with more than one chronic condition might need support to coordinate their care arrangements. This is reflected by the choice for quality of care as perceived by the patient as the main outcome measure. When case management superposed on diabetes management is more effective than diabetes management alone, many patients will benefit from a better organized care, better health outcomes, and more efficient use of health care services. From a societal perspective, effective case management will lead to more efficient health care utilization, and consequently lower costs.

The major strength of this study is the close fit to the normal procedure in primary care practice and the not invasive character of the case management program. Experiments with case management as proposed in this study are a logical next step in pursuing optimal care for diabetics with comorbidity, with the knowledge that more than 60% of the diabetics have one or more comorbid disorders. Also, when people get older, the prevalence of chronic illnesses increases. Taking this into account, care for comorbid diabetics can become very complex to manage. The model of case management superposed on disease management for diabetics can be seen as exemplary and it will be worthwhile to apply the results - if positive - to other single-disease management programs for other chronic diseases.

The designed program is based on the Guided Care Model which is under study in the USA. There are three major differences between the implementation of the case management program in the USA and the customized version in our study.

At first, we do not screen for eligible patients based on their health care consumption (or costs) or based on frailty measures. We have chosen to include a group of patients already enrolled in a diabetes management program, who are diagnosed with multiple chronic conditions. Because they have multiple chronic conditions, they could participate in several disease management programs. These programs aim at structuring and managing all care necessary according to the evidence-based guidelines for the specific chronic disease. However, these programs are not designed to complement each other and could increase burden and costs for the patients, especially when there are multiple comorbidities present. For that reason the question whether case management has any additional value over the structured diabetes management and if so, for which patients with comorbidity, is very important. This is also a core subject of the ZonMw research program on disease management, who provide funding for this study.

Secondly, the case manager in our study is less extensively involved in coordinating the patients' care than in the USA and for example does not coordinate transitions between sites (from hospital back to home or vice versa). When a patient is in hospital, the case manager will be informed by the patient, family or hospital. The case manager will contact the patient when he or she is still in hospital or after discharge to discuss and, if necessary, adjust the care plan. Participants in this study are not institutionalized. They are motivated and empowered during this intervention to contact the case manager if problems or changes in health status occur. Important is the emphasis on patient priorities and patient self management, monitored by monthly telephone contacts, which also implies that patients are responsible for keeping the case manager up-to-date.

Thirdly, we do not include training for the patient's informal caregivers, because the participants in this study are expected not to be severely frail. We are aware of the burden informal caregivers often face and therefore they are welcome to accompany their spouses during their participation in the case management program.

This study had some limitations in the design that might influence the reliability and validity of this study, which we have to address.

Our study population is a standardized group of T2DM patients already receiving structured diabetes care in a diabetes management program.

This could influence the external validity of the study. However at this moment the majority of the T2DM patients in the Netherlands are included in a disease management program, which means that structured diabetes management is more or less 'usual care' in primary care. Therefore we expect our study results to be generalizable, meaning it can be translated to a larger group of comorbid diabetics in the Netherlands.

We look at the additional value of the case management program next to an existing disease management program. This means that using a selected standardized population has its benefits. The patient's health is monitored every 3 months at the GP and the information in the medical records should be up-to-date.

We identify possible participants by reviewing the electronic medical record at the general practice, using disease specific (ICPC) codes. Mental illnesses such as depression are often not well recognized or diagnosed in primary care. We might miss out on patients with this condition. We expect that most participants in this study will receive the case management program tailored to somatic disorders.

In this study we trained two Practice Nurses to conduct the program. They will include 230 participants in this study, which means a caseload of 115 participants per person - half enrolled in the intervention group and half receive usual care. These participants are divided over multiple primary care practices. Differences in results between participants managed by the case managers can occur. To avoid bias due to the number of case managers we will check the data for cluster-effect by case manager.

The case managers work together with the PN and GP to discuss the results from the initial assessment and during the design of the care plan before it is presented to the patient. During this contact the GP and PN might get motivated to use specific elements in the usual care group, who do not receive the intervention. This could lead to contamination. However, the GP and PN are not involved in the program (the actual problem assessment with the patients, the monthly monitoring, the contacts between the case manager and participant) and therefore we expect this form of contamination, if it occurs, will be washed out in a short period and will not influence the intervention effect after 12 months.

This study started in October 2009 by designing the program and making all practical arrangements for the start and implementation of the intervention. In February 2011 the first patients were included in the study. Follow-up of patients will continue until July 2012.

The results of this study will become available at the beginning of 2013.

## Abbreviations

CQ-Index: Consumer Quality Index for GP care; DCS: Diabetes Care System; GC: Guided Care Model; GP: General Practitioner; HbA1c: glycated haemoglobin level; ICPC: International Classification of Primary Care; PACIC: Patient Assessment of Chronic Illness Care; PCAT: Primary Care Assessment Tool; PN: Practice Nurse; RAI-CHA: Resident Assessment Instrument - Community Health Assessment; SF-12: Short-Form 12; T2DM: type 2 Diabetes Mellitus.

## Competing interests

The authors declare that they have no competing interests.

## Authors' contributions

FGS and GN designed the study. NV drafted the article and all authors contributed to the final concept. All authors have read and approved the final manuscript.

## Pre-publication history

The pre-publication history for this paper can be accessed here:

http://www.biomedcentral.com/1471-2296/12/68/prepub
